# The Mediator and/or Moderator Role of Complexity of Knowledge about Healthy Eating and Self-Regulated Behavior on the Relation between Family’s Income and Children’s Obesity

**DOI:** 10.3390/ijerph16214207

**Published:** 2019-10-30

**Authors:** Beatriz Pereira, Pedro Rosário, Cátia Silva, Gabriela Figueiredo, José Carlos Núñez, Paula Magalhães

**Affiliations:** 1 Department of Applied Psychology, Escola de Psicologia, Universidade do Minho, Campus Gualtar, 4710-052 Braga, Portugal; beatriznpereira94@gmail.com (B.P.); prosario@psi.uminho.pt (P.R.); catiasbsilva@gmail.com (C.S.); gsscfigueiredo@hotmail.com (G.F.); 2 Department of Psychology, Universidad de Oviedo, Plaza Feijoo s/n. 33003 Oviedo, Spain; jcarlosn@uniovi.es; 3 Facultad de Ciencias Sociales y Humanidades, Universidad Politécnica y Artística de Paraguay, Mayor Sebastián Bullo s/n, Asunción 1628, Paraguay

**Keywords:** elementary school age, BMI Z-scores, complexity of knowledge about healthy eating, family’s income, self-regulation, SOLO taxonomy

## Abstract

Childhood obesity rates have been increasing over the years and is considered one of the most serious public health problems of the century. Low socioeconomic status has been associated with a higher body mass index. However, the pathways underlying this complex relationship are poorly understood. This study aimed to evaluate the possible mediation and/or moderation effects of complexity of knowledge about healthy eating, and self-regulation processes towards healthy eating, in the association between family’s income and weight of elementary school age children. The results showed that complexity of knowledge does not mediate the relationship between socioeconomic status and weight. Still, whenever the levels of complexity of knowledge and self-regulation are high, there is a moderation effect of complexity of knowledge on the relationship between family’s income and weight, conditioned by self-regulation scores. These promising findings support the idea that knowledge about healthy eating in isolation could be insufficient for practicing a healthy diet and underline the relevance of combining transmission of knowledge with training in specific competences (e.g., self-regulation strategies). Considering that complexity of knowledge about healthy eating and self-regulation strategies are interrelated and can be both improved, future preventive interventions could consider incorporating both in their design to mitigate childhood obesity.

## 1. Introduction

Obesity is considered a chronic, relapsing progressive, and growing epidemic disease [1]. In fact, obesity prevalence has been increasing to alarming levels. According to the World Health Organization [2], obesity rates went up three times worldwide since 1975. Concerning childhood obesity, the scenario is as daunting and, thus, considered one of the most serious public health challenges of the 21st century [3]. For example, data from Portugal shows that 28.5% of children between the ages of 2 and 10 are overweight, with 12.7% being obese [4]. Along with health problems, there are psychological, social, and economic consequences associated with child and adult obesity [5,6,7,8,9]. Regarding health, there are many problems strongly related to childhood obesity, such as new onset of asthma, cardiovascular diseases, type 2 diabetes, and certain types of cancer [7,8]. Additionally, over an extensive period, childhood obesity is associated with increased risk of adult obesity and increased morbidity and mortality in adult life [7]. Regarding psychological and social consequences, obesity is associated with low self-esteem, behavioral problems, and social isolation [8]. Furthermore, obesity also has an impact on the economy. For example, obese adults spend annually $US 3508 more in medical costs than those who maintain a healthy weight [5], and is estimated that around 7% of national health budgets across the EU are spent on diseases linked to obesity each year [6].

Finding solutions to this scenario requires understanding its etiology. Obesity and overweightness are the results of a combination of multiple factors, such as genetic, environmental, and individual [10,11,12]. Although there are many elements contributing to childhood obesity, research shows that most cases are associated with non-genetic and modifiable factors, such as eating habits and physical activity [13,14]. However, for children in low-income families, eating habits do not seem easily modifiable. In fact, parents with low socioeconomic status (SES) are less likely to purchase healthy food or to demonstrate healthy dietary behaviors [15]. The limited financial resources and the high cost of healthier diets may contribute to this shortness of healthy food purchases [16,17]. In fact, fats and sweets provide dietary energy at significantly low expense, whereas eating vegetables and fruit leads to an increase in dietary costs [18]. Conversely, families with high SES, in addition to fewer budget restrictions to purchase healthy food, show more effective parental investments in child’s health. Additionally, they tend to be integrated into school and community environments that promote physical exercise and healthy eating practices [19,20]. Consequently, there is a negative relationship between SES and obesity that seems to be associated with children’s eating habits and influenced by food parental practices at home, i.e., low SES is associated with higher body mass index (BMI) and this relationship is likely to strengthen over time [21,22,23,24,25,26]. 

Concurrently, research shows that parents with low SES have lower nutritional knowledge (e.g., understanding the health benefits such as disease prevention) than parents with higher SES, and parents’ nutritional knowledge directly predicts children’s knowledge about nutrition [27]. Thus, children from low SES households whose parents possess low nutritional knowledge are likely to have low nutritional knowledge themselves. Research shows that a lack of knowledge about what is healthy to eat is a major challenge in nutritional education [28,29]. To illustrate, 70% of EU individuals believe their diets are already healthy and do not know how to evaluate their own diet appropriately in terms of fat, fibers, fruits, and vegetable intake [29]. Furthermore, studies show positive associations between nutritional knowledge and likelihood of healthy food consumption, indicating that nutritional knowledge is necessary for making healthy food choices [30,31]. 

Considering this evidence, it comes as no surprise that most of the interventions to tackle obesity and overweightness have focused on transmitting knowledge about healthy eating. For example, interventions emphasize the increased consumption of vegetables and fruits and the regular practice of physical exercise to decrease the likelihood of becoming obese [32]. However, despite the considerable investment in knowledge-based interventions and programs for children, in a wide range of settings, there has been no substantial improvement in healthy eating, and childhood obesity does not seem to be reverting [33,34]. In fact, a metanalysis with several interventions showed heterogeneity on nonsignificant improvements in healthy eating regardless of the modality of the intervention (e.g., cognitive vs. non-cognitive, healthy eating only vs. combined lifestyle intervention), with an exception for interventions with reinforcement [33]. A possible underlying cause for the ineffectiveness of these interventions is that research shows that beliefs about healthy eating (e.g., think that broccolis is good) have a stronger influence on eating behavior than declarative knowledge [35]. That is, by itself knowledge about healthy eating is not a guaranty of healthy food choices. 

A recent systematic review suggests that, among other aspects, using self-regulation strategies in the implementation of a healthy diet may be key for individuals to adopt healthy eating habits [36]. Self-regulation comprises processes that allow individuals to proactively control the personal, behavioral, and environmental influences that impact human behavior [37]. For instance, various studies have shown that the extent to which people engage in planning behaviors to reach a goal is associated with healthy or unhealthy food intake [38]. Hence, understanding the role of self-regulation in behavior change and the adoption of healthy eating habits may be key to develop and design effective programs [39]. 

**Figure 1:**
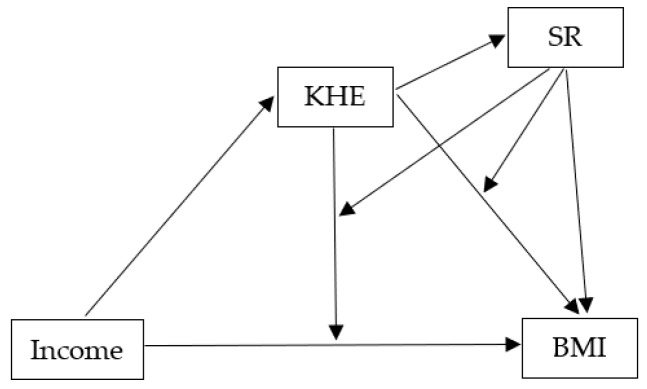
Conceptual Model of mediational and moderator role of the complexity of knowledge about healthy eating (KHE) on the relation of family’s income and BMI Z-scores, and the mediator/moderator role of self-regulation (SR) in the KHE and BMI Z-scores relationship.

Most studies on factors that contribute to obesity prevention evaluate variables in isolation. However, an examination of combinations of factors may be required to improve a comprehensive understanding of the complex obesity prevention phenomenon [36]. All things considered, the main aim of the present study was to evaluate the possible mediational and/ or moderation effects of complexity of knowledge about healthy eating (KHE) and self-regulation (SR) processes towards healthy eating in the association between family’s income and BMI Z-scores of elementary school age children (see Figure 1). Elementary school students were chosen as participants of this study as previous research has suggested that this age is the ideal stage to discuss, learn, and enact behavior change, including in healthy eating domain [40,41,42]. Considering the aim of contributing to understanding how reverting the increasing rate of childhood obesity, participants of the present study had a weight range between healthy and obese (BMI Z-scores). This methodological option allowed to understand how KHE and SR may contribute to decrease obesity and promote a healthy BMI on elementary school age children. 

Grounded on previous research, a mediation-moderation model (Figure 1) was set with the following hypothesis:

**H1.**  *There is a statistically significant relationship between Family’s Income and BMI. The higher the Family’s Income the more likely they are in the healthy BMI range, and the lower the Family’s Income the more likely the children are overweight and obese.*

**H2.**  *The relationship between Family’s Income and BMI is mediated by KHE. It is expected that the higher the Family’s Income the better their KHE, and the more children in the healthy BMI range.*

**H3.**  *The relationship between KHE and BMI maybe mediated by SR. That is, the more complex the participants’ KHE, the higher their SR and the higher their SR, the more the proximity with a healthy BMI range. This means that the effect of more complexity of knowledge about healthy eating on children’s BMI is likely to occur when they self-regulate their food intake behaviors.*

**H4.**  *Moreover, besides the mediation effect, the relationship between KHE and BMI is possibly moderated by SR. It is expected that the intensity and direction of the relationship between KHE and BMI are conditioned by the SR scores. It is expected this relation to be more intense when SR scores are higher.*

**H5.**  *The relationship between Family’s Income and BMI is moderated by KHE. The magnitude of this relationship is significantly different for distinct KHE scores.*

**H6.**  *The relationship between Family’s Income and BMI is moderated by KHE and this moderation effect is conditioned by the SR scores.*

## 2. Materials and Methods 

### 2.1. Participants

The participants were selected from five schools in the North of Portugal. This study appertains to a large research project (CEICSH 032/2019) counting with 744 students from the 5th and 6th grades and their parents. Participants had already agreed to participate and provided written informed consent. Of the initial pool of participants, 445 (59%) had to be excluded of the present study due to: 1) at baseline parents did not provide all the necessary information for BMI Z-scores calculation (e.g., date of birth of their child, weight), 2) students did not complete all the measures collected (e.g., did not answer to the open-ended question). Additionally, 4 (1.3%) participants were excluded due to their BMI Z-score being >-1SD (e.g., thinness). Considering the aims of the present study, i.e., contribute to understanding how to mitigate overweightness and obesity and promote healthy weight, these participants were excluded as they do not provide information helpful to attain these goals. Thus, the final sample was composed of 295 students who were healthy weight, overweight (>+1SD), and obesity (>+2SD).

### 2.2. Instruments

#### 2.2.1. BMI Z-Scores 

To evaluate children’s BMI, the Z-score classification system was used [43]. The Z-score system expresses the anthropometric value as a number of standard deviations below or above the reference mean or median value. This is widely recognized as the best system for analysis and presentation of anthropometric data because of its advantages compared to the other methods (e.g., percentiles) [44]. For example, the Z-score scale is linear and therefore a fixed interval of Z-scores has a fixed height difference in cm, or weight difference in kg, for all children of the same age, which makes results comparable across ages groups and indicators.

In the present study, parents/caregivers were asked about their child’s height, weight, and date of birth. To calculate children’s Z-scores according to children’s height, weight, gender, and age in months, the *WHO AnthroPlus* software was used. The classification through z-scores for five-19 years is: Obesity: >+2 *SD* (coded for the current study as 1), Overweight: >+1 *SD* (coded as 2), Healthy range weight: between +1 *SD* and −1 *SD* (coded as 3), Thinness: between −1*SD* and −2 *SD* and Severe thinness: >−2*SD*.

#### 2.2.2. Family’s Income

Income is a simple indicator that aims to capture one’s ability to purchase desired resources [45]. Specifically, financial resources have an important influence on food purchases [16,17].

To evaluate the family’s income, students’ School Social Action Level was requested. In Portugal, the School Social Action corresponds to the following three levels of family income (i.e., sum of the income of the whole household members): A, corresponds to a family’s income of up to 3050.32 € per year, B, corresponds to a family’s income of up to 6100.64 € per year, and C, corresponds to a family’s income of up to 9150.96 € per year. An income superior to this last value corresponds to a family that does not have School Social Action, is considered a high income [46]. It should be considered that the Portuguese minimum wage in 2019 is 600 € per month and that a worker who earns the minimum wage has a gross remuneration of 8400 € per year [47]. Accordingly, in the present study Family’s Income was classified as 1 = extremely very low income, 2 = very low income, 3 = low income, 4 = medium to high income.

#### 2.2.3. Complexity of Knowledge About Healthy Eating 

To evaluate the complexity of knowledge about healthy eating, the open-ended format question “What is healthy eating?” was presented to the participants. An open-ended question format allows to reduce the likelihood of biased responses by the participants and is better suited to capture participants’ knowledge, diversity of conceptions, and beliefs about a topic compared with closed-format questions [48]. 

Responses to the question were classified through the Structure of the Observed Learning Outcome (SOLO) Taxonomy [49]. SOLO Taxonomy evaluates the complexity/quality of an answer’s structure about a concept (comprehension complexity). The five levels represent the five different possible ways for an individual to structure an answer (from incompetence to expertise) and is applicable to any subject area. In fact, it is a powerful tool for qualitative assessment of the point at which children are currently in in their understanding of a concept. 

In the present study, to classify the responses of the participants to the open question, researchers developed a codebook for each level of complexity of the SOLO taxonomy (see Table 1). This codebook addressing each level of the SOLO taxonomy was developed by taking the writing samples produced by the participants who did not enter in the final sample of the present study (see Section 2.1). For example, Biggs and Collis [49] describe the unistructural level as “the learner focuses on the relevant domain and picks one aspect to work with”. On the present research codebook, the relevant domain is healthy eating and one aspect could be not to drink sugary drinks.

Next, prior to the coding of the participants’ responses, two researchers trained the coding scheme as follows: (i) researchers first discussed the distinguishing features of each of the codebook levels, (ii) researchers practiced together applying the scale to a series of open-ended answers that varied in terms of their complexity until agreed on coding, (iii) each researcher independently scored 10 open-ended answers during practice, compared scores and resolved any differences through discussion, (iv) training continued until they assigned scores that differed by no more than 1 open-ended answer on ten consecutive ones. Once the criteria were met, one researcher scored all of the open-ended answers written by the participants in this study and the other researcher independently scored 25% of that open-ended answers. Interrater reliability between the two researchers was 0.947.

#### 2.2.4. Self-Regulation Processes towards Healthy Eating Questionnaire 

An adapted version of the Self-Regulation for Health Scale [50] was used to evaluate the self-regulation processes towards healthy eating that participants are guided by. The recorded internal consistency was of 0.77, value which has been obtained using Cronbach’s Alpha coefficient on the present study. The scale has nine statements regarding the participants’ self-regulation towards healthy eating (e.g., I pay attention to information on healthy eating, I apply that knowledge on a daily basis). Responses are scored from 1 (never) to 5 (always) on a Likert-like scale and summed to create a composite score from nine to 45, with higher scores implying more self-regulation. 

### 2.3. Procedure

The present study is part of a research project that has been approved by the University of Minho Ethics Committee for Research in Social and Human Sciences (CEICSH) (CEICSH 032/2019). CEICSH considered that the project complies with the requirements for good practice in human research in accordance with national and international standards governing research in social and human sciences, including the Declaration of Helsinki. Additionally, consent to conduct the study was obtained from the Portuguese Ministry of Education. Prior to data collection, written informed consent from children and parents/caregivers were requested. To protect confidentiality and anonymity of the data, codes were assigned to identify the participants.

Data collection took place in a regular class. Students were invited by a research assistant to fill in the previously mentioned instruments and the research assistant highlighted the need to complete the task individually. Children took approximately 25 min to complete the questionnaires.

### 2.4. Statistical Analysis

The statistical examination of the mediation-moderation model (see Figure 1) was approached as follows. First, descriptive analyses (mean, standard deviation, skewness, and kurtosis) and Pearson correlations were conducted. Secondly, to analyze the hypothesis, several regression analyses were done using PROCESS from SPSS 22 [51]. Mediation, moderation, and conditional analysis were conducted. The effect sizes were calculated using Cohen’s (1988) *d* statistic (*d* < 0.20 = non-significant, *d* ≥ 0.20 and *d* < 0.50 = small effect, *d* ≥ 0.50 and *d* < 0.80 = medium effect, *d* ≥ 0.80 = large effect).

## 3. Results

In the present study participated 295 students with ages comprised between 9 and 14 years (*M* = 10.89, *SD* = 0.849), of which 54.8% were female.

### 3.1. Descriptive Statistics

Table 2 presents descriptive statistics and the Pearson correlation matrix. Skewness and kurtosis data indicate that the variables follow a normal distribution. Moreover, statistically significant relationships were found between Family’s Income and BMI (the higher the Family’s Income the more proximal BMI will be to the healthy weight range), between Family’s Income and KHE (the higher the Family’s Income the more the complexity of knowledge about healthy eating), and between KHE and SR (the higher the KHE the higher the scores on SR). The remaining correlations were not statistically significant (e.g., SR and Family’s Income).

### 3.2. Mediation, Moderation and Conditional Analysis

The current research examines a set of models to explain the relationships between the Family’s Income and their children’s BMI Z-scores (from now onwards just BMI). First, the mediation processes were examined as follows: (i) analyze how the Family’s Income affects their children’s BMI. To this purpose, the role of KHE as mediator in the relationship between Family’s Income and BMI was analyzed. Moreover, (ii) it was also analyzed the mediator role of SR between KHE and BMI. Additionally, the moderator role of KHE and SR (i.e., the boundary conditions for an association between two variables) was investigated. In fact, it was studied (iii) how the relationship between Family’s Income and BMI was moderated by KHE, and (iv) how the relationship between KHE and BMI was moderated by SR. In this process, (v) both the indirect as well as the interactive effects (i.e., conditional process analysis, or second stage moderation model [52]) were analyzed. Specifically, it was addressed how the relation between Family’s Income and BMI was mediated by KHE and, at the same time, how the relationship between KHE and BMI was moderated by SR. Finally, (vi) it was analyzed how the potential moderator role of KHE could be conditioned by SR (this last step aimed to answer the following question: Is the effect of Family’s Income on BMI Z-scores affected by the scores of KHE, but only for certain scores of SR?).

Table 3 presents data from the fit of the six models tested to address the hypotheses raised.

In general, data gathered from the fit of the models do not confirm the hypothesis set. However, findings support, despite marginally, the first hypothesis (i.e., there is a statistically significant relationship between Family’s Income and BMI: *b* = 0.0580, *p* = 0.0898, Model 1, Table 3). The second hypothesis was not confirmed, data indicates that the indirect effect of Family’s Income on BMI trough KHE, was not statistically significant (*b* = 0.0090, *p* = 0.1912). The no relationship found between KHE and BMI (*b* = 0.0877, *p* = 0.1280, see Model 1, Table 3) may help explain the latter. Findings allow us to conclude that KHE is not mediating the relationship between Family’s Income and BMI. 

To further understand the absence of direct effect of KHE on BMI we set the third hypothesis, to examine whether this effect could be not direct, but indirect through SR. Data do not support the third hypothesis, i.e., no indirect effect of KHE on BMI was found (note that the relation between SR and BMI is not statistically significant, *b* = −0.0333, *p* = 0.6192). Still, data also show that by including SR in the model the direct effect of KHE on BMI is significant (*b* = 0.1101, *p* = 0.0501, see Model 2 in Table 3). In sum, data indicate that KHE mediates the effect of Family’s Income on BMI when SR is included in the model (see Model 2 in Table 3). 

The inclusion of SR improved the model, so the next step was to analyze the role of this variable as a moderator of the effect of KHE on BMI (hypothesis 4). The aim was to examine whether the strength or direction of this relationship was conditioned by the scores of SR (see Model 4, Table 3). It was hypothesized that the higher the scores of SR the more intense the relationship between KHE and BMI would be. Data from the first stage moderation model (see Model 4, Table 3) (*b* = 0.0025, *p* = 0.9739) and from the second stage moderation model (see Model 5, Table 3) (*b* = 0.0165, *p* = 0.8321) indicates that SR is not moderating the effect of KHE on BMI. 

In sum, considering current data from the fit of the six models presented thus far, SR is not mediating nor moderating the effect of KHE on BMI, but when included in the model as a potential mediator, KHE partially mediates the effect of Family’s Income on BMI. Note that, besides analyzing the mediator role of KHE in the relationship between Family’s Income and BMI, it was also investigated the moderator role of KHE on this relationship (see Model 3, Table 3). At that stage, it was hypothesized that the magnitude of the relationship between Family’s Income and BMI varies depending on the scores of KHE (hypothesis 5). Data indicates that the relationship between Family’s Income and BMI is not conditioned by the students’ level of complexity of knowledge on healthy eating (KHE × Income = 0.0464, *p* = 0.3435, see Model 3 Table 3), thus the increment on the explained variance of BMI due to the inclusion of KHE as moderator was not statistically significant (R^2^ change = 0.0030). However, when analyzing the conditional effect of Family’s Income on BMI at the values of KHE, data indicates that for higher scores on KHE the effect of Family’s Income on BMI is marginally statistically significant (*b* = 0.0909, *p* = 0.0624). 

Considering the aforementioned findings, a new model (Model 6 Table 3) was set to investigate whether the absence of moderation of KHE (for low and medium scores) on the effect of Family’s Income on BMI could be related to the moderator effect of SR (hypothesis 6). It was hypothesized that the relationship between Family’s Income and BMI is moderated by KHE when this moderation effect is conditioned by the scores of SR (see Model 6, Table 3). The results of the model fit have partially confirmed this hypothesis. Table 4 shows that the interaction (Income × KHE × SR) is marginally significant (*b* = 0.1061, *p* = 0.0933) and increments significantly the explained variance of BMI (R^2^ change = 0.0094, *F* (1,291) = 2.8358, *p* = 0.0933). However, the effect of Family’s Income on BMI is only statistically significant for KHE and SR high scores (see, Table 4). Additionally, data show that for low SR, the effect of Family’s Income on BMI does not vary irrespective of the KHE scores, and also that the higher the SR and the KHE scores, the stronger the effect of Family’s Income on BMI.

## 4. Discussion

Childhood obesity is one of the most serious public health challenges of the 21st century [3]. The etiology of obesity and overweightness is complex and multifactorial, being the result of a combination of genetic, environmental, and individual factors [10,11,12]. To decrease this worrying scenario, it is important to deepen the knowledge of how modifiable factors influence obesity and overweightness, especially among children from families with low income. Literature has been stressing the importance of knowledge about healthy eating and self-regulation, but data on this topic is limited. Thus, the present study aimed to understand the mediation and/or moderation role of complexity of Knowledge about Healthy Eating (KHE) and Self-Regulation (SR) on the relationship between Family’s Income and their children’s BMI Z-scores (BMI). 

First, results showed that there is a relationship between Family’s Income and BMI. Higher Family’s Income is associated with a BMI more proximal to the healthy weight range and lower Family’s Income is associated with overweightness and obesity. This promising finding is consistent with previous research. Studies have shown that there is a negative relationship between SES and obesity [21,22,23]. In fact, a systematic review concluded that low SES, including low income, is the only factor that has so far witnessed consistent evidence for unhealthy eating and risk of overweightness and obesity [36]. 

Second, data shows that in the present study KHE does not serve as a mediator of the effect of Family’s Income on BMI (see Model 1 in Table 3). In fact, KHE is related to Family’s Income but not to BMI. This result allows us to conclude that it cannot be argued that, by itself, a greater or lesser complexity of knowledge about healthy eating is a crucial variable for the control of obesity. Research shows that parents with low SES have lower nutrition knowledge, and that parent’s low nutrition knowledge predicts five and six years-old children’s low nutrition knowledge [27]. However, current data alerts to the fact that providing knowledge about healthy eating in isolation could be insufficient to improve healthy eating and revert overweightness and obesity. For example, previous studies show that whereas information about healthy diet is available to consumers, it is often regarded as complicated and difficult to implement, even if consumers have apprehended the knowledge conveyed [36]. Altogether, our findings may help understand why, despite the considerable investment in interventions and programs, there has been no substantial improvement in healthy eating and obesity does not seem to be reverting [33,34]. 

Third, it was explored the mediating and moderating role of SR. The rationale for analyzing this step was that the effect (weak or strong) of the complexity of knowledge on children’s BMI could be related to the level of SR. For example, children with high complexity of knowledge about healthy eating but with low SR competences to follow a healthy diet could show overweightness as well as children with low complexity of knowledge, but strong SR competences. Surprisingly, results show that SR does not mediate nor moderate the relationship between KHE and BMI (see Model 2 and 4 in Table 3). Instead, data shows that the conditional effect of KHE on the Family’s Income relationship with BMI was, in turn, conditioned by the levels of SR (see Model 6 in Table 3). Current results showed that moderation only occurs for high levels of KHE and SR. This promising finding may contribute to further understand the complex relationship between Family’s Income and BMI. Data indicates that Family’s Income influences BMI whenever the levels of KHE and SR are high (i.e., children from high Family’s Income can be obese if they do not have deep complexity of knowledge about healthy eating and do not display self-regulated behaviors, and children from low Family’s Income can have healthy weight range if they have deep complexity of knowledge about healthy eating and self-regulate their health behaviors). All considered, these promising findings support the idea that knowledge about healthy eating in isolation could be insufficient for practicing a healthy diet [36]. The formula for mitigating childhood obesity does not seem to be the promotion of a single factor in isolation, i.e., neither promoting knowledge in isolation is enough, nor promoting self-regulation in isolation is enough. Instead, it seems that the promotion of knowledge about healthy eating combined with self-regulation strategies may be a promising approach for supporting healthy eating behaviors. 

Thus, the design of future interventions may wish to focus not only on transmitting knowledge on healthy eating habits but combining it with the training on self-regulated strategies related to healthy eating behaviors. For example, design programs with hands-on activities to exercise PLEE (planning, execution, evaluation) logic in meal preparation or to practice choice and control skills towards food purchase is likely to promote healthy eating behaviors [37,39,53]. We believe that the promising findings of the present study could be used in educational settings in different ways: (a) through curricular infusion - teachers may use their classes as opportunities to embed self-regulation strategies into their curricula and work the contents simultaneously. For example, when teaching the food wheel in the Sciences discipline, teachers can use activities of meal planning. In doing so, teachers meet the curricular goals while promoting reflection about how children can use the declarative knowledge to make healthier choices. Thus, children do not receive information about healthy eating passively but are expected to reflect on the process [37]. (b) through school-based programs—school administrators may organize educational interventions running outside school hours to transmit knowledge about healthy eating and promote self-regulated strategies. Moreover, interventions based on the promotion of knowledge about healthy eating combined with self-regulation strategies could also be used to train parents. Parents play an important role in transmitting knowledge to their children and parent support is an important facilitator in healthy eating [27,54,55]. For example, the family could influence children’s early-life experiences with various tastes and flavors [55]. Thus, it is important that parents provide their children accurate knowledge and opportunities to use self-regulation strategies at home.

As our data suggest, it is important to work “healthy food choices” at the individual level. However, individuals’ daily lives and their more or less healthy eating habits are influenced and shaped by economic and social policies, and social norms and developmental agendas that must also be acknowledged [56,57]. The outcomes of the current research are expected to be used by advocacy groups comprised of children, parents, teachers, and the health community to advocate for the importance of launching policies on healthy eating habits. In fact, to promote healthy eating habits and facilitate individual healthy food choices, we need to consider social determinants of health and use multiple strategies. For example, prior literature highlights the importance of promoting children’s health by supporting policies that restrict aggressive marketing encouraging sedentary activities, energy-dense, and nutrient-poor foods, and unhealthy beverages [56,57]. Advocacy groups for healthy eating habits could consider working with the governmental agencies and food companies to help set environmental and structural changes likely to facilitate individual healthy choices (e.g., improve health literacy, encourage low-cost price leadership models, advertise healthy choices as optimal choices). The work done by these advocacy groups over the individual and social determinants of health is expected to generate public debate on this issue and, hopefully, help flourish innovative obesity prevention strategies. 

Although the present research entails an advancement in the understanding of the moderating effect of KHE and SR in the relationship between Family’s Income and BMI, some limitations of the study and suggestions for future research should be addressed. Despite the effect of Family’s Income on BMI being statistically significant, the effect size is minimal. One explanation for that could be the measurement of the variables: (i) the information for the BMI Z-scores calculation was provided by the parents, instead of being measured directly by the researchers. This could add self-report bias, i.e., instead of report children’s actual weight/height, parents may report the last values that they remember or values more proximal to the normative standards, (ii) Family’s Income could not be considered a continuous variable because, although the first three levels could be continuous, the fourth level is something else, being that more income intervals could be considered in the fourth category (e.g., 9000 to 12000 €, 12000–15000 €), (iii) the use of Family’s Income instead of compound variable to define socioeconomic status, iv) knowledge about healthy eating could also be defined in many different ways. In the present study, the SOLO taxonomy allowed to transform qualitative data into a continuous quantitative variable to make possible run mediation and moderation models [51]. However, while measuring the complexity of knowledge we may have lost content information. Future research could consider analyzing knowledge using a different methodological approach (e.g., thematic analysis to the open-ended question). Another limitation of the study refers to its design. Due to the cross-sectional nature of the study, a causal relationship regarding associations between the variables could not be established [51]. Future research should consider a longitudinal design to provide a methodological advantage to infer the causal relationship between the variables. Moreover, despite promising, the effect sizes of the present study results are small. Finally, although it is not possible to guarantee the generalization of the present findings, current data were collected in five Portuguese public schools from different environments (e.g., rural, urban). This variety of settings allows a wide range of socioeconomic and cultural backgrounds. Consistently, different factors that influence overweightness and obesity have been studied for decades, but the impact of these determinants on actual eating behavior has been shown to be small to medium [36]. In fact, many studies on factors likely to influence healthy eating and obesity focus on one or two factors in isolation, while insight in particular combinations of factors may be required to improve a comprehensive understanding of the drivers of healthy eating [36]. For example, future research could consider studying the role of variables such as self-efficacy and attitudes towards healthy eating and use complex analysis designs combining mediation and moderation effects conjointly. Thus, it seems important to continue studying the relationships between different modifiable variables in order to understand how to design preventive interventions to decrease this worldwide complex problem. 

## 5. Conclusions

Overall, the results of the present study contribute to the existing literature, shedding light on the complex relationships between Family’s Income and BMI. To further understand this relationship, we tested the mediational role of KHE, but results were not statistically significant. Further analysis testing a moderation role of KHE on the relationship between Family’s Income and BMI showed positive results when this moderation effect is conditioned by the scores of SR. These findings are expected to provide an important reference to the design of future preventive interventions on eating behaviors. Considering that knowledge about healthy eating and self-regulation strategies are interrelated and can be improved, interventions could consider incorporating both in their design. Finally, future research could consider study other possible mediators/moderators that may have a significant influence on the relationship between Family’s Income and BMI of children. For example, motivational variables related to volition (strengthen children will to follow a healthy diet) but also metacognitive variables related with children awareness of the pros and cons of following a healthy diet. 

## Figures and Tables

**Table 1:** SOLO coding criteria applied to complexity of knowledge about healthy eating.

Level	Name	Descriptors	Examples
1	Prestructural	1. reveals lack of effort and engagement; 2. does not understand the question; 3. repeats the question; 4. mentions something irrelevant; 5. wrong answers.	“I don´t know”; “It is to eat healthy things”.
2	Unistructural	1. reveals minimum effort; 2. the answer is correct, but it only focuses on one aspect.	“It is to have balanced eating habits”; “It is not to eat chocolate”.
3	Multistructural	1. knows some content; 2. describes, lists; 3. more aspects are perceivable, however not correlated; 4. connects sentences using “and”, “also”; 5. answers in which two or more elements are mentioned and connected, however, this correlation is wrongfully made.	“It is to always drink water, not to drink too many sodas and not to eat too much candy”; “It is to eat vegetables and fruits because they are carbohydrates”.
4	Relacional	1. applies knowledge, recognizes these applications; 2. connects concepts in a way that the whole text has a coherent and meaningful structure; 3. gives an explanation, justifies.	“It is to eat food which is good for your health, like lettuce, tomatoes, kale…”; “It is not to eat too much candy or sugar, as we could get diabetes.”
5	Extended Abstract	1. reflects, evaluates, formulates; 2. takes into consideration all the relevant information (and may also consider information which is not part of the task) and its connections, lists them and deducts a hypothesis that may be applied to a different situation; 3. there is generalization.	“It is to eat soup, fruit and vegetables every day, not to eat too much candy (although sometimes it is allowed), not to consume sparkling beverages… if we don’t follow a healthy diet, we may suffer serious consequences in our growth. Healthy eating is very important”.

**Table 2:** Descriptive statistics and Pearson correlation matrix.

Variables	Income	BMI	KHE	SR
Family’s Income	−			
Body Mass Index (BMI)	0.115 *	−		
Complexity of Knowledge about Healthy Eating (KHE)	0.173 **	0.106	−	
Self-Regulation (SR)	−0.014	−0.008	0.191 **	−
*M*	2.970	2.448	2.920	3.824
*SD*	1.215	0.709	0.721	0.623
Asimetria	−0.569	−0.895	−0.360	−0.534
Kurtosis	−1.349	−0.506	1.292	0.065
Mínimum	1	1	1	1
Máximum	4	3	5	5

Note: Family’s Income (1 = extremely very low income; 2 = very low income; 3 = low income; 4 = medium to high income); BMI (1 = obesity; 2 = overwheight; 3 = healthy weight range); KHE (1 = prestructural; 2 = unistructural; 3 = multistructural; 4 = relational; 5 = extended abstract); SR (1 = very low SR SR; 2 = low SR; 3 = moderate SR; 4 = high SR; 5 = very high SR). * *p* < 0.05. ** *p* < 0.01.

**Table 3:** Results of the Mediation, Moderation, and Conditional Process Analysis.

Model	Description of Model	Effects	Coeff.	se	t	*p* <	d
1	Mediation Model: Effect of Family’s Income on BMI mediated by KHE	Income → KHE	0.1027	0.0339	3.0281	0.0027	0.36
		KHE → BMI	0.0877	0.0575	1.5261	0.1280	−
		Income → BMI	0.0580	0.0341	1.7022	0.0898	0.20
2	Mediation Model: Effect of KHE on BMI mediated by SR	KHE → SR	0.1652	0.0493	3.3532	0.0009	0.40
		SR → BMI	−0.0333	0.0669	−0.4975	0.6192	−
		KHE → BMI	0.1101	0.0579	1.9015	0.0501	0.22
3	Moderation Model: Effect of Family’s Income on BMI moderated by KHE	KHE → BMI	−0.0453	0.1515	−0.2992	0.7650	−
		Income → BMI	−0.0781	0.1475	−0.5294	0.5969	−
		KHE × Income → BMI	0.0464	0.0489	0.9488	0.3435	−
4	Moderation Model: Effect of KHE on BMI moderated by SR	SR → BMI	−0.0404	0.2264	−0.1784	0.8585	−
		KHE → BMI	0.1006	0.2949	0.3412	0.7332	−
		SR × KHE → BMI	0.0025	0.0776	0.0328	0.9739	−
5	Second Stage Moderation Model: Effect of Family’s Income on BMI mediated by KHE and the effect of KHE on BMI moderated by SR	Income → KHE	0.1027	0.0339	3.0281	0.0027	0.36
		KHE → BMI	0.0307	0.2969	0.1034	0.9177	−
		Income → BMI	0.0581	0.0344	1.6877	0.0925	0.20
		SR → BMI	−0.0737	0.2266	−0.3255	0.7450	−
		SR × KHE → BMI	0.0165	0.0778	0.2123	0.8321	−
6	Moderation-Moderated Model: Effect of Family’s Income on BMI conditioned by the interaction of KHE and SR	KHE → BMI	0.9227	1.8376	0.0735	0.9414	−
		Income → BMI	0.8770	0.6772	1.4640	0.1443	−
		SR → BMI	0.5955	0.5016	1.1873	0.2361	−
		Income × KHE →BMI	−.3580	0.2378	−1.5055	0.1333	−
		Income × SR → BMI	−0.2554	0.1848	−1.3771	0.1696	−
		KHE × SR → BMI	−0.2757	0.1818	−1.5167	0.1304	−
		Income × KHE × SR → BMI	0.1061	0.0630	1.6840	0.0933	0.20

Note: Family’s Income (Income), Body Mass Index (BMI), Complexity of Knowledge about Healthy Eating (KHE), Self-Regulation (SR).

**Table 4:** Conditional effect of socioeconomic status of family on student’ Body Mass Index at values of the moderators SR (Self-Regulation) and KHE (Complexity of Knowledge about Healthy Eating).

Self-Regulation	Complexity of Knowledge about Healthy Eating	Effect	se	t	*p* <	d
3.2005	2.1954	0.0218	0.0571	0.3827	0.7023	—
3.2005	2.9164	0.0084	0.0531	0.1592	0.8736	—
3.2005	3.6374	−0.0050	0.0785	−0.0630	0.9498	—
3.8241	2.1954	0.0084	0.0530	0.1581	0.8745	—
3.8241	2.9164	0.0427	0.0354	1.2062	0.2287	—
3.8241	3.6374	0.0770	0.0510	1.5088	0.1324	—
4.4477	2.1954	−0.0051	0.0780	−0.0650	0.9482	—
4.4477	2.9164	0.0769	0.0490	1.5698	0.1175	—
4.4477	3.6374	0.1589	0.0628	2.5291	0.0120	0.30
